# Identification of a Pathogenic *TGFBR2* Variant in a Patient With Loeys–Dietz Syndrome

**DOI:** 10.3389/fgene.2020.00479

**Published:** 2020-05-27

**Authors:** Xi Luo, Shan Deng, Ying Jiang, Xiang Wang, Abdulrahman Mustafa Ahmed Al-raimi, Long Wu, Xiaobin Liu, Yu Song, Xiao Chen, Feng Zhu

**Affiliations:** ^1^Department of Cardiology, Wuhan Union Hospital, Tongji Medical College, Huazhong University of Science and Technology, Wuhan, China; ^2^Clinic Center of Human Gene Research, Wuhan Union Hospital, Tongji Medical College, Huazhong University of Science and Technology, Wuhan, China; ^3^Department of Cardiovascular Surgery, Wuhan Union Hospital, Tongji Medical College, Huazhong University of Science and Technology, Wuhan, China

**Keywords:** aorta dissection, aneurysms, Loeys–Dietz syndrome, transforming growth factor beta receptor 2, transforming growth factor β

## Abstract

Loeys–Dietz syndrome (LDS) is a rare connective tissue genetic disorder that is caused by a pathogenic variant in genes of transforming growth factor (TGF) beta receptor 1 (*TGFBR1*), *TGFBR2*, mothers against decapentaplegic homolog 2 (*SMAD2*), *SMAD3*, *TGFB2*, or *TGFB3*. It is characterized by aggressive vascular pathology, aneurysms, arterial tortuosity, bifid uvula, hypertelorism, and cleft palate. Here we present a 42-year-old female patient with LDS. The patient underwent rapidly progressing artery aneurysms and life-threatening aortic dissection. Spontaneous fracture of the first metatarsal bone was noted in her medical record. Physical examination revealed a delayed wound healing on her left abdomen. Considering these clinical manifestations, we speculated that there was a genetic defect in the connective tissue, which provides strength and flexibility to structures such as bones, skins, ligaments, and blood vessels. Thus, whole exome sequencing (WES) was performed on the proband and revealed a heterozygous missense pathogenic variant (c.1613T > C/p.Val538Ala) in *TGFBR2*, which was a *de novo* variant in the proband as confirmed by the segregation analysis in parental samples. Although this variant was discovered and associated with the phenotype of LDS previously, the pathogenicity of the variant had not been confirmed by cellular functional assay yet. To further validate the effects of the variant *in vitro*, we assessed the canonical TGF-β signaling pathway in mutant cells. Our results showed that the p.Val538Ala variant significantly decreased TGF-β-induced gene transcription and the phosphorylation of Smad2, which were consistent with other pathogenic variants of *TGFBR2*. In conclusion, this study demonstrates that the p.Val538Ala pathogenic variant in *TGFBR2* leads to aberrant TGF-β signaling and LDS in this patient.

## Introduction

Loeys–Dietz syndrome (MIM#609192, LDS) is an inherited autosomal dominant connective tissue disorder with a broad phenotypic spectrum of cardiovascular, skeletal, craniofacial, and cutaneous manifestations (Loeys et al., 2005, 2006). Transforming growth factor (TGF) beta receptor 1 (TGFBR1) and 2 (TGFBR2) are serine/threonine kinase receptors, which can activate downstream mothers against decapentaplegic homolog (SMAD) signaling cascades after ligand binding. Numerous studies have shown that TGF-β signaling pathway regulates various critical cellular processes such as cell proliferation, differentiation, angiogenesis, and matrix transformation; and pathogenic variants in genes involved in this pathway are the major cause for the pathogenesis of LDS (Regalado et al., 2011; Boileau et al., 2012; Bertoli-Avella et al., 2015).

The pathologic features of LDS are elastin degradation and cystic medial necrosis in the connective tissues, resulting in rapidly progressive aortic aneurysmal disease in LDS patients (Maleszewski et al., 2009). Clinical features are overlapping with other genetic connective tissue disorders, such as Marfan syndrome (MIM#154700, MFS) and Ehlers–Danlos syndrome (MIM#130050, EDS). Some LDS patients were previously diagnosed with MFS due to the similarities in symptoms, but the cardiovascular manifestations in LDS are much more significant. Compared to MFS, dilatation of the aortic root can lead to aortic dissection and rupture at much smaller diameters and younger ages in patients with LDS (Takeda et al., 2018). Therefore, treatment regimen and prognosis are vastly different between LDS and MFS. Patients with LDS require close monitoring and early treatment to extend the life span. Genotyping of patients presenting with symptoms like artery aneurysm and dissection may be used to guide therapy, including timing of vascular surgery (MacCarrick et al., 2014).

In this study, we reported a case of a 42-year-old female who underwent recurrent and life-threatening artery aneurysm and dissection, spontaneous bone fracture, and delayed wound healing. Genetic test revealed a *de novo* heterozygous missense pathogenic variant (c.1613T > C/p.Val538Ala) in *TGFBR2*, which was implicated as a potential cause for the pathogenesis of LDS (Luo et al., 2016). Additionally, we performed cellular functional assays and demonstrated that the c.1613T > C/p.Val538Ala variant disrupted TGF-β signaling pathway.

## Case Presentation

**FIGURE 1:**
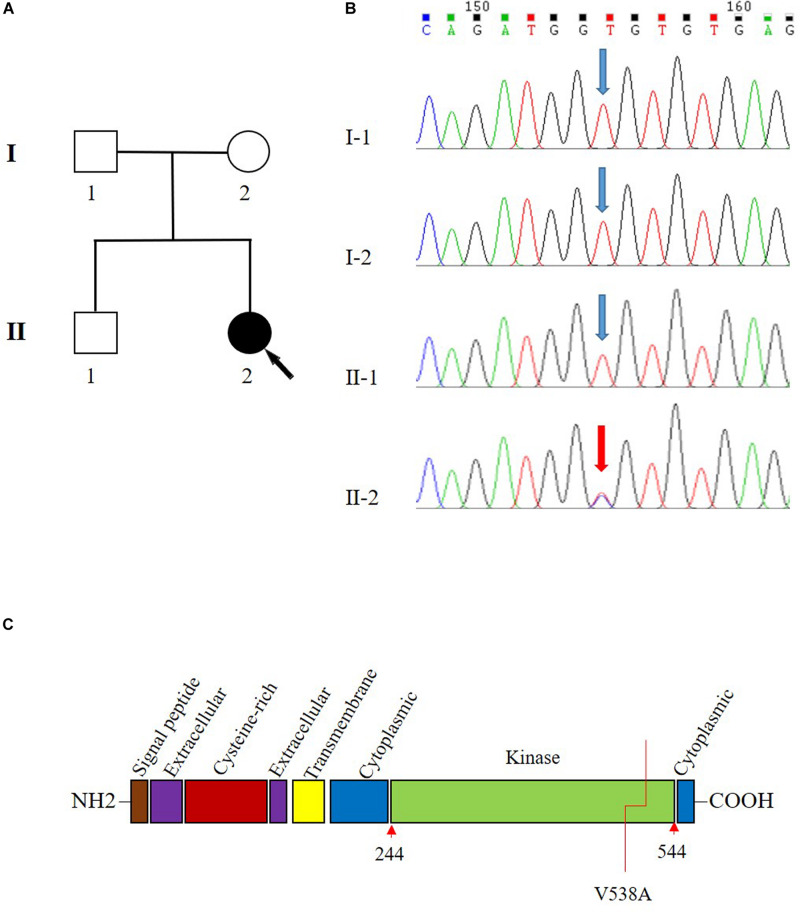
Pedigree and Sanger sequencing chromatogram of c.1613T > C (p.Val538Ala) variant in the transforming growth factor beta receptor 2 (*TGFBR2*). **(A)** Family pedigree. Black arrow indicates the proband, II-2. **(B)** The variant T1613 > C in TGFBR2 is identified in II-2 (proband). Blue arrow indicates wild type in I-1, I-2, and II-1. The reference sequence NM_001024847.2 of *TGFBR2* is used. **(C)** The domain structure of *TGFBR2* and V538 is located in the kinase domain.

**FIGURE 2:**
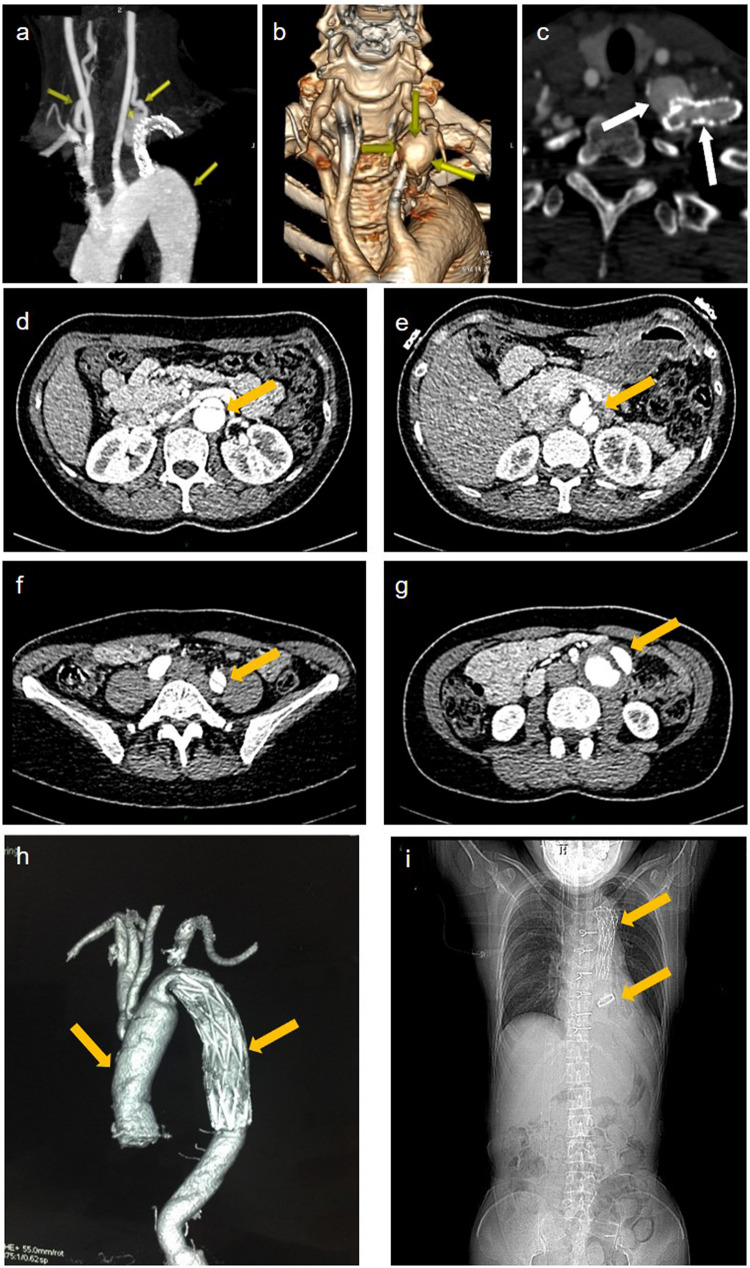
Imaging data of the patient. **(a)** representative maximum intensity projection picture of the left subclavian artery which has been stented to exclude the aneurysm. **(b)** volume rendered (vr) three-dimensional (3d) reconstructed images show a large aneurysm of the left subclavian artery near its origin from the aortic arch. **(c)** the cross section showed an opacification of the aneurysm with contrast agent seen with the parent vessel indicating an endoleak. **(d–f)** aortic ct angiography found debakey type iii dissecting aortic aneurysm ranged from the opening of the celiac trunk **(e)**, the proximal rupture located at the level of the bilateral renal artery **(d)**, and ended at the left internal iliac artery **(f)**. **(g)** the false lumen of the lower abdominal aorta showed aneurysmal dilatation and mural thrombosis. **(h)** the endovascular stent–graft placement was shown. **(i)** x-ray showing thoracic incision and metal valves after aortic valve replacement.

A 42-year-old female patient (II-2) was admitted to our department complaining of dizziness and chest tightness. Her parents (I-1 and I-2) and sibling (II-1) were healthy (Figure 1A). They all belong to the Han ethnicity in Wuhan, Hubei, China, without family history of any genetic disease. Her medical records showed rapidly progressive and widespread arterial tortuosity. She was diagnosed with subclavian artery aneurysm with a diameter of 10.2 mm at the age of 34 and received the subclavian artery stent implantation because of the increasing size of aneurysm (Figure 2a). But a large aneurysm (18.1 mm × 33.1 mm) of the left subclavian artery near its origin was still visible in three-dimensional (3D) reconstructed images from computed tomography angiography (CTA) 2 years after the stent implantation (Figures 2b,c). Later on, she suffered from aortic dissection at the age of 37. CTA revealed DeBakey type III dissecting aortic aneurysm, of which the proximal rupture was located at the level of the bilateral renal artery (Figure 2d). The dissection ranged from the opening of the celiac trunk to the left internal iliac artery (Figures 2e–g). Then she received endovascular stent–graft placement (Figure 2h). At the age of 40, she suffered from aortic root and arch aneurysms again and underwent Bentall operation (aortic root replacement) with total aortic arch replacement as well as elephant trunk repair surgery (Figure 2i). In addition, she had a history of a first metatarsal fracture without external force damage at the age of 38. On physical examination, the patient had a normal stature at a height of 163 cm without spinal deformity (Figure 2i). There was no significant craniofacial dysmorphism. Mild anemia was evident with a hemoglobin concentration of 99 g/l hemoglobin. The patient had an incurable skin wound on her left abdomen after the elephant trunk repair surgery. Given that the patient had developed arterial tortuosity, spontaneous fracture, and delayed skin wound healing, we hypothesized that she was likely suffering from a genetic connective tissue disease, such as MFS, EDS, or LDS.

## Identification of Pathogenic Variants

To systematically search for the gene variants associated genetic connective tissue disease, whole exome sequencing (WES) was performed on the patient. The mean sequencing coverage on target regions was 76.8-fold, providing enough data to obtain 99.19% at 20 × coverages of 39 Mb targeted exome of the human genome (hg19). Based on the aligned reads, 64,227 initial variants (57,092 SNVs, 7135 indels) were identified. The filtering cascades for WES data are listed in Supplementary Table S1. After five filters of the variants data for WES data, 347 variants from 267 genes were kept. These genes were then associated with the phenotype of “aortic dissection; artery aneurysm” by Phenolyzer, and the result revealed one heterozygous T-to-C transition c.1613T > C in *TGFBR2* (Supplementary Figure S1), which leads to a substitution of valine to alanine at codon 538 (p.Val538Ala) in the *TGFBR2* kinase domain (Figure 1C). This variant is a raw variant which is absent in population databases including Genome Aggregation Database (gnomAD), Exome Aggregation Consortium (ExAC), Exome Sequencing Project (ESP), and 1000 Genomes. The evaluation of possible functional impacts revealed that c.1613T > C/p.Val538Ala was classified as a damaging pathogenic variant by SIFT (score = 0.004), MutationTaster (score = 1), clinPred (score = 0.88), and possible damaging by Polyphen2 (score = 0.802) (Shihab et al., 2013). Since all functional prediction tools produce false negatives, the known pathogenic variants related to aortic dissection may be ruled out following our filtering process. To identify the known pathogenic variants which might be excluded, we generated a list containing the variants in 28 known disease-causing genes that might cause aortic dissection (Pinard et al., 2019) to identify the known pathogenic variants according to Clinvar database (Supplementary Excel S1). There were no more known pathogenic or likely pathogenic variants in the disease-causing genes other than the *TGFBR2* gene. We also analyzed all detected variants related to genetic cardiovascular disorders according to the American College of Medical Genetics and Genomics (ACMG) statement of secondary findings in clinical exome and genome sequencing (Kalia et al., 2017). We identified two variants of uncertain significance, c.2020G > A/p.Glu674Lys in *KCNQ1* and c.12878C > T/p.Ala4293Val in *RYR1* according the 2015 ACMG/Association for Molecular Pathology (AMP) Standards and Guidelines for the interpretation of sequence variants (Richards et al., 2015). But neither of these genes was medically associated with aortic dissection based on current knowledge (Treves et al., 2005; Hedley et al., 2009).

**FIGURE 3:**
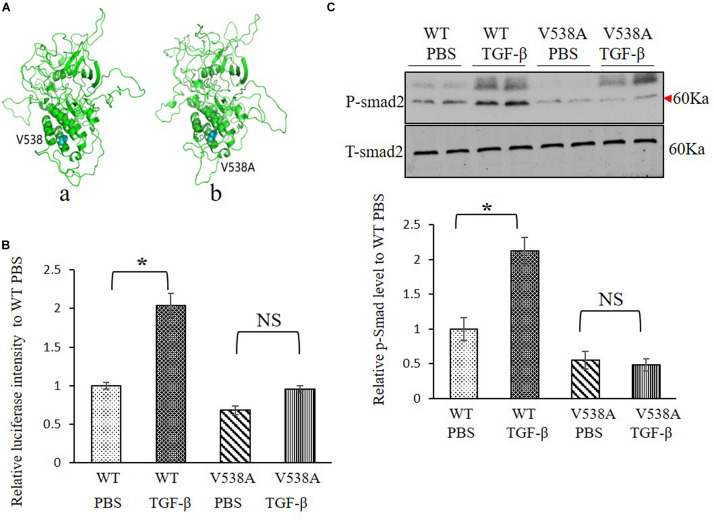
Transforming growth factor (TGF) beta receptor 2 (TGFBR2) V538A has decreased canonical TGF-β signaling *in vitro*. **(A)** The native **(a)** and mutated **(b)** structure of TGFBR2 after molecular dynamics (MD) simulation. **(B)** Luciferase reporter assay showed that overexpression of TGFBR2 V538A in TGFBR2-deficient HCT116 cells led to decreased TGF-β-associated gene transcription following TGF-β1 stimulation. *N* = 3, ^∗^*p* < 0.05 versus wild-type (WT) phosphate buffered saline (PBS) group. **(C)** Representative Western blotting pictures and quantification demonstrated lower phosphorylation levels of mothers against decapentaplegic homolog 2 (SMAD2) in TGFBR2 V538A in TGFBR2-deficient HCT116 cells following TGF-β1 treatment when compared with WT. *N* = 4, ^∗^*p* < 0.05 versus WT PBS group.

Molecular structure differences between *TGFBR2* c.1613T > C/p.Val538Ala mutant protein and wild-type (WT) protein were investigated *in silico*. Modeling was performed by using the software of MODELLER in a rough model. Two protein structures (Protein Data Bank accession code: 5e92 and 3q4t) were used as homology models. Then, a short time molecular dynamics (MD) simulation by AMBER software was employed to evaluate the molecular stability. The overall scaffold of the mutant structure is similar to the WT as shown in Figure 3A. However, there is much difference between coiled regions which is instinctively disordered.

Sanger sequencing analysis identified c.1613T > C/p.Val538Ala only present in the patient (II-2) while absent in her unaffected parents (I-1 and I-2) and sibling (II-1) (Figure 1B). Further paternity test using multiplex short tandem repeat typing (DC8902, Promega) confirmed the biological relationship between the patient and her parents (Supplementary Table S2), thus confirming the *de novo* nature of the variant. Additionally, we found that this variant was absent in 200 normal controls, who all were healthy Han people in Wuhan, excluding c.1613T > C/p.Val538Ala as a rare polymorphism.

### Cell-Based Functional Assays Demonstrate That the c.1613T > C/p.Val538Ala Variant Displays Aberrant Activity

To investigate the impact of the variant c.1613T > C/p.Val538Ala on TGF-β signaling pathway, a luciferase reporter assay was performed, and the phosphorylation of SMAD2 level was measured to determine the downstream transcriptional activation. We constructed two plasmids expressing WT or mutant TGFBR2 protein. For the reporter assay, we bought the luciferase reporter construct containing a TGF-β responsive element that drives luciferase expression and co-transfected it with WT or c.1613T > C/p.Val538Ala mutant plasmids into HCT116, which is refractory to TGF-β-mediated signaling due to biallelic frameshift mutations in the A10 coding microsatellite of the endogenous TGFBR2 (Lee et al., 2013). Results showed that, comparing with WT TGFBR2, transcriptional activation in the mutant TGFBR2 is noticeably suppressed with or without TGF-β1 stimulation (Figure 3B). In accordance with this result, Western blotting also showed that phosphorylation of SMAD2 in the mutant TGFBR2 group is reduced compared with WT upon stimulation with TGF-β1 (Figure 3C). These results confirm the hypothesis that the p.Val538Ala substitution impairs the function of TGFBR2 and disrupts the TGF-β signaling pathway.

### Clinical Interpretation of the c.1613T > C/p.Val538Ala of the *TGFBR2* Gene

According to the 2015 ACMG/AMP, the missense pathogenic variant on c.1613T > C/p.Val538Ala was classified as “pathogenic.” Detailed evidence-based information is shown in Table 1.

**TABLE 1:** Clinical interpretation of genetic variants by ACMG/AMP 2015 guideline.

Variant	Variant type	Variant classification	Criteria*	Strength of the criteria
c.1613T > C;	missense	Pathogenic	PS2	Strong
p.Val538Ala			PS3	Strong
			PM1	Moderate
			PM2	Moderate
			PP3	Strong

*^∗^PS2, *de novo* (both maternity and paternity confirmed) in a patient with the disease and no family history; PS3, well-established *in vitro* or *in vivo* functional studies supportive of a damaging effect on the gene or gene product; PM1, located in a mutational hot spot and/or critical and well-established functional domain (e.g., active site of an enzyme) without benign variation; PM2, absent from controls (or at extremely low frequency if recessive) in Exome Sequencing Project, 1000 Genomes Project, or Exome Aggregation Consortium; PP3, multiple lines of computational evidence support a deleterious effect on the gene or gene product (conservation, evolutionary, splicing impact, etc.); ACMG, American College of Medical Genetics and Genomics; AMP, Association for Molecular Pathology.*

## Discussion

In the present study, we described the clinical features of a Chinese woman who was diagnosed with LDS. Although the genetic test identified a reported heterozygous missense pathogenic variant on c.1613T > C/p.Val538Ala of the *TGFBR2*, we first evaluated the pathogenicity of this variant *in vitro*. Our cellular functional assays demonstrated that this *TGFBR2* variant can significantly inhibit TGF-β–Smad signaling pathway and was a pathogenic variant.

Loeys–Dietz syndrome is a congenital disorder with an abnormal increase of collagen and extracellular matrix caused by activated TGF-β signal pathway. It affects the connective tissue in many parts of the body, especially, skeleton, heart, blood vessel, and skin. Six different genes involved in this disease have been found so far, which are classified as types I–V of LDS. In 2005, the pathogenic variants in genes *TGFBR1* and *TGFBR2* were first identified as causes of LDS, which was called Type I and Type II, respectively. Subsequently, pathogenic variants in *SMAD3* (Type III) (Regalado et al., 2011), *TGFB2* (Type IV) (Boileau et al., 2012), and *SMAD2* and *TGFB3* (Type V) (Bertoli-Avella et al., 2015) were also reported to be pathogenic variants associated with LDS. This disorder is characterized by the triad of arterial tortuosity and aneurysms, hypertelorism, and bifid uvula or cleft palate. The natural history is characterized by aggressive arterial aneurysms and a high rate of pregnancy-related complications. Types I and II LDS appear to be the most common forms. Data suggested that the onset of vascular disease occurred at significantly younger age among the Type 1 cohort compared to the Type 2 cohort, but individuals with *TGFBR2* pathogenic variants were more likely to dissect at aortic diameters less than 5.0 cm than individuals with *TGFBR1* variants (Tran-Fadulu et al., 2009). Here, we discovered that this patient had the c.1613T > C/p.Val538Ala variant of *TGFBR2*, which belongs to LDS Type II, and the clinical manifestation is consistent with LDS. However, diagnosis of LDS was delayed due to the absence of significant skeletal abnormalities and characteristics in this patient. Nonetheless, the genetic test provided strong evidence supporting the diagnosis of LDS. Impressively, using luciferase assay with HCT116 cells, we validated the inhibitory effects of c.1613T > C/p.Val538Ala variant on TGF-β-associated transcriptional activation, and it reduced phosphorylation of SMAD2 after stimulation with TGF-β *in vitro*, which were all consistent with previous findings (Horbelt et al., 2010; Cousin et al., 2017). Since the c.1613T > C/p.Val538Ala variant of *TGFBR2* was reported as a likely pathogenic variant in 2016 (Luo et al., 2016), these cellular results confirmed the pathogenicity of this variant.

Previous variants about *TGFBR2* mutant reported in ClinVar showed that 86 of the 87 plausible pathogenic missense variants are located in the kinase domain. Study showed that p.Val419Leu variant in the *TGFBR2* affected the receptor function through alteration of its structure and inactivation of kinase conformations (Cousin et al., 2017). In this study, we discovered that the c.1613T > C/p.Val538Ala variant also located in the kinase domain of TGFBR2 protein, and the overall scaffold of the mutated structure is similar to the native one by MD stimulation. However, there is much difference between coiled regions which is instinctively disordered. Considering the patient’s symptom and results from *in vitro* experiments, we anticipate that this variant will affect the kinase activity of the receptor and disrupt the downstream signal transduction. Yet, future studies on the structure and the kinetics of conformation change of the mutant receptor should be warranted.

Intuitively, gene mutation-associated diseases are caused by loss of function of the encoded protein (Doyle et al., 2012; Franken et al., 2013). Surprisingly, as shown in many clinical case reports, the TGF-β signaling pathway was activated in LDS patient tissue samples (Cousin et al., 2017; Hara et al., 2019). Although the underlying detailed pathogenic mechanisms for this phenomenon remain largely unknown, it might be related to compensatory activation of a non-canonical pathway (Sorrentino et al., 2008; Iwata et al., 2012) and maladaptation of negative feedback mechanism (Lindsay and Dietz, 2011). Recently, one study postulated that the regional microenvironment and specifically lineage-dependent variation in the vulnerability to mutations are important factors governing the activation of TGF-β signaling pathway (MacFarlane et al., 2019). Further mechanistic studies are needed to resolve this problem.

There are several limitations in our study. First, only blood samples were used to evaluate the gene variants; tissue samples would be better to directly assess TGF-β signaling in the patient. Second, due to technical limitations, the detailed structure and kinetics of conformation change for this variant could not be analyzed, and we were unable to rule out the presence of possible mosaicism of the variant without high-throughput sequencing. Third, we did not put a known pathogenic variant in TGFBR2 for comparison which would make our results more solid.

In conclusion, our study reports a *de novo* pathogenic variant c.1613T > C/p.Val538Ala of the *TGFBR2* gene in a Chinese woman with LDS. As the clinical prognosis of LDS is poor and the treatment is urgently needed for patients, our study can significantly aid in the diagnosis of complex or special cases.

## Materials and Methods

### Subjects

The Ethical Committee of the Tongji Hospital, Tongji Medical College, Huazhong University of Science and Technology, Wuhan, China, reviewed and approved our study protocol. All participants as well as parents of underage patients have given written informed consent.

### Genetic Testing

Whole exome sequencing was performed by using xGen Exome Research Panel v1.0 (IDT, United States) on the Illumina Novaseq platform. According to the manufacturer’s protocol, genomic DNA was extracted from whole blood, then sheared by sonication, and hybridized for enrichment. After the library was enriched for target regions, sequencing was performed to generate 150-bp paired-end reads. To identify pathogenic variants on the proband, sequencing data were analyzed and annotated according to an in-house pipeline. Briefly, raw reads were preprocessed to remove reads with low quality or adapters. Then, clean reads were mapped to the human reference genome (GRCh37) using Burrows–Wheelers Aligner (BWA, version 0.7.8-r455) (Li and Durbin, 2009). The generated bam file was sorted by SAMtools (Li et al., 2009). SAMtools (version 1.0) was performed to call single nucleotide variants (SNVs) and indels (<50 bp), while CoNIFER (Krumm et al., 2012) was applied to detect copy number variations (CNVs). After that, ANNOVAR (Wang et al., 2010) accompanied with several prediction tools were used for annotating SNVs, indels, and CNVs. Notably, each variant was compared against several public databases, including 1000 Genomes Project^1^, NHLBI ESP 6500^2^, ExAC (ExAC release 0.3.1^3^), and gnomAD^4^ (Lek et al., 2016) to achieve allele frequency. In terms of a possible influence on the protein function, variants were evaluated by several popular prediction tools: Sorting Intolerant from Tolerant (SIFT) (Ng and Henikoff, 2003), Polymorphism Phenotyping version 2 (PolyPhen-2) (Adzhubei et al., 2010), MutationTaster (Schwarz et al., 2010), ClinPred (Alirezaie et al., 2018), and Genomic Evolutionary Rate Profiling (GERP++) (Davydov et al., 2010). To identify the known pathogenic variants, the detected variants were compared against the Clinvar database^5^. Based on the variant annotations, a series of prioritization strategies were applied to identify candidate variants associated with the phenotypes. The detailed steps were as follows: (1) excluding variants outside exonic and splicing regions; (2) excluding variants with minor allele frequency (MAF) > 0.01 according to public databases; (3) excluding synonymous variants; (4) excluding non-conservative variants with score ≤ 2 according to GERP++conservation prediction; (5) excluding variants not presenting damaging results in any protein function prediction from SIFT, Polyphen2, MutationTaster, and ClinPred. Remaining data after the five steps formed a list of candidate variants and related genes. To prioritize the most likely candidate disease-causing gene, all candidate genes were then ranked by Phenolyzer^6^ (Yang et al., 2015), a tool using prior information to implicate genes involved in diseases. “Aortic dissection” was input as a phenotype term into Phenolyzer.

To confirm the candidate disease-causing variant, we PCR-amplified the genomic DNA fragments in all familial members, then sequenced them by Sanger sequencing. The primers used in PCR are: forward 5’–CAGGCACTCAGTCAGCACAT–3’and reverse 5’–TTCCTGCTGCCTCTGTTCTT–3’.

The pathogenicity of the identified variants was evaluated according to the 2015 ACMG/AMP Standards and Guidelines (Richards et al., 2015; Li and Wang, 2017).

### Cell Culture

HCT116 cells were cultured in Dulbecco’s modified Eagle’s medium (DMEM) supplemented with 10% fetal bovine serum (FBS), and 1% penicillin/streptomycin at 37°C and 5% CO_2_. When the cells grow to about 70% density, different plasmids were transferred as designed.

### Luciferase Reporter Assays

Plasmids pCDNA3.1-TGFBR2-WT (WT) and pCDNA3.1-TGFBR2-Mut (with variant p.Val538Ala) were constructed in Tsingke Biological Technology Company. Plasmid p3TP-Lux, a luciferase reporter construct containing a TGF-β responsive element driving luciferase expression was bought on addgene (#11767). When the confluency of HCT116 in six-well plate was about 60%, we cotransfected plasmid p3TP-Lux with WT or mutant TGFBR2 using Lipofectamine 3000 (Thermo Scientific). Twenty-four hours later, cells were treated with 5 ng/ml TGF-β1 (PeproTech, #100-21) in medium without serum for an additional 24 h. At last cells were collected to measure luciferase expression. Total protein concentration was used to control luciferase activity, and all conditions were normalized to unstimulated cells overexpressing WT TGFBR2. Four independent experiments were completed with *t*-tests indicating significance between conditions.

### Western Blotting

After treatment, cells were collected and lyzed in radioimmunoprecipitation assay (RIPA) buffer containing 0.1 mM phenylmethylsulfonyl fluoride (PMSF), protease inhibitor cocktail (Roche), and phosphatase inhibitors (Thermo Fisher Scientific). After quantification and denaturation, each sample was loaded with equal protein (30 μg), separated by 12% sodium dodecyl sulfate–polyacrylamide gel electrophoresis and transferred to polyvinylidene fluoride membrane. After being blocked with 5% non-fat milk in Tris-buffered saline (TBS) for 1 h, the membranes were incubated at 4°C overnight with primary antibodies of total SMAD2 (1:1,000; Cell Signaling 5339S), pSMAD2 (1:1,000; Cell Signaling 3101S). On the following day, membranes were incubated in horseradish peroxidase (HRP)-conjugated secondary antibody for 1 h, and the specific bands were detected by super ECL reagent (Pierce, Rockford, IL, United States) and developed with super ECL reagent (Pierce, Rockford, IL, United States). ImageJ was used to quantify Western blot density.

## Data Availability Statement

All datasets generated for this study are included in the article/Supplementary Material.

## Ethics Statement

Family members of this patient have given written informed consent as they are participating in this study. The Ethical Committee of the Union Hospital, Tongji Medical College, Huazhong University of Science and Technology, Wuhan, China, reviewed and approved our study protocol in compliance with the Helsinki declaration.

## Author Contributions

XL and SD collected the clinical data, analyzed the data, and wrote the manuscript. YJ and AA did the cellular experiments. XW, LW, XBL, and YS edited the manuscript. XC designed the cellular experiments. FZ supervised and conceptualized the study and edited the manuscript.

## Conflict of Interest

The authors declare that the research was conducted in the absence of any commercial or financial relationships that could be construed as a potential conflict of interest.

## Footnotes

1 https://www.internationalgenome.org/

2 http://evs.gs.washington.edu/EVS/

3 http://exac.broadinstitute.org

4 https://gnomad.broadinstitute.org

5 https://www.ncbi.nlm.nih.gov/clinvar

6 http://phenolyzer.wglab.org/
