# Supplementary material for: Identification of a Pathogenic TGFBR2 Variant in a Patient With Loeys–Dietz Syndrome
Source: Front Genet. 2020 May 27;11:479. doi: 10.3389/fgene.2020.00479 (PMC7266969; doi:10.3389/fgene.2020.00479)

**Supplemental Table 1. The number of variants after each filtering step**

| **Step** | **variants** |
| --- | --- |
| Initial | 64,226 |
| 1.excluding variants outside exonic and splicing regions | 27,669 |
| 2.excluding variants with minor allele frequency (MAF) >0.01 | 943 |
| 3. excluding synonymous variants | 679 |
| 4. excluding non-conservative variants with score ≤ 2 according to GERP++ conservation prediction | 523 |
| 5. excluding variants not presenting damaging results in any protein function prediction from SIFT, Polyphen2, MutationTaster and ClinPred | 347 in 267 genes |
| 7. Phenolyzer (term “aortic dissection”) | 1 |

**Supplemental Table 2. Multiplex short tandem repeat typing for paternity test**

| Gene loci | father | Proband | mother |
| --- | --- | --- | --- |
| D19S433 | 15, 15 | 9.2, 13 | 9.2, 15 |
| D5S818 | 12, 14 | 12,14 | 11, 12 |
| D21S11 | 29, 31.2 | 31.2 , 31.2 | 28, 31.2 |
| D18S51 | 16, 17 | 16, 19 | 11, 14 |
| D6S1043 | 19, 19 | 14, 19 | 11, 19 |
| D3S1358 | 17, 17 | 15, 17 | 15, 17 |
| D13S317 | 10, 12 | 10, 11 | 11, 11 |
| D7S820 | 10, 11 | 9, 11 | 9, 10 |
| D16S539 | 9, 11 | 9, 11 | 9, 12 |
| CSF1PO | 11, 14 | 12, 14 | 12, 14 |
| Penta D | 9, 11 | 9, 11 | 11, 13 |
| AMEL | X , Y | X , X | X , X |
| vWA | 14, 17 | 14, 15 | 15, 17 |
| D8S1179 | 10, 11 | 10, 15 | 13, 15 |
| TPOX | 8, 9 | 8, 10 | 8, 10 |
| FGA | 24, 25 | 24, 25 | 23, 24 |
| PentaE | 11, 14 | 11, 16 | 13, 16 |
| TH01 | 6, 9 | 9, 10 | 9, 10 |
| D2S1338 | 18, 20 | 20, 24 | 23, 24 |
| D1S1656 | 15, 16 | 16, 17 | 16, 16 |

The allele of the patient can be found from the alleles of his parents. The cumulative parental authority indexes are 141989985.5018 for father and 1113684339.1849 for mother.

**Supplemental Figure 1. Top 20 genes ranked by Phenolyzer according to their associations with term “aortic dissection”.**


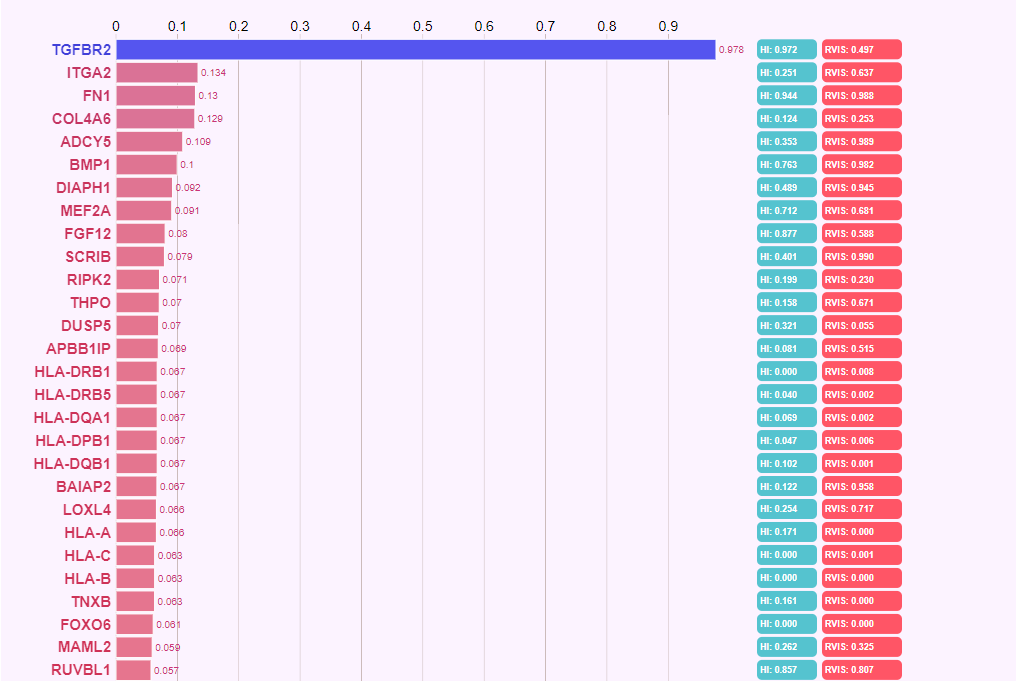

Supplement: Supplementary file 2 [file Data_Sheet_2.docx]
